# Ionic Disorders in Persistent Atrial Fibrillation: Therapeutic Targeting by Plant‐Derived Bioactive Compounds

**DOI:** 10.1155/crp/2123244

**Published:** 2026-07-09

**Authors:** Yize Zhai, Na Shi, Wenzhuo Duan, Jianfei Yang

**Affiliations:** ^1^ Heilongjiang University of Chinese Medicine, Harbin, China, hljucm.edu.cn; ^2^ The First Affiliated Hospital of Heilongjiang University of Chinese Medicine, Harbin, China, hljucm.edu.cn

**Keywords:** arrhythmia, cardiac electrophysiology, ion channel dysfunction, persistent atrial fibrillation, plant-derived bioactive compounds

## Abstract

Persistent atrial fibrillation (AF) is associated with distinct ion channel abnormalities and their regulatory mechanisms. This review explores the potential of plant‐derived bioactive compounds to correct these dysfunctions. As a prevalent cardiac arrhythmia, AF involves a complex pathogenesis featuring dysregulated activity in potassium, sodium, and calcium channels. Such ionic disturbances foster electrophysiological instability in cardiomyocytes, which contributes to AF initiation and sustenance. Plant‐based compounds have attracted considerable attention for their multitargeted actions, low toxicity, and biocompatibility. Emerging evidence indicates that specific phytochemicals can modulate ion channel function and improve myocardial electrical stability, offering potential avenues for AF therapy. However, the precise mechanisms and clinical translational potential of these compounds require further elucidation. By systematically synthesizing current knowledge, this review assesses the value of plant‐derived compounds in rectifying ionic imbalances, with the aim of informing the development of novel antiarrhythmic therapies and future research directions.

## 1. Introduction

**FIGURE 1:**
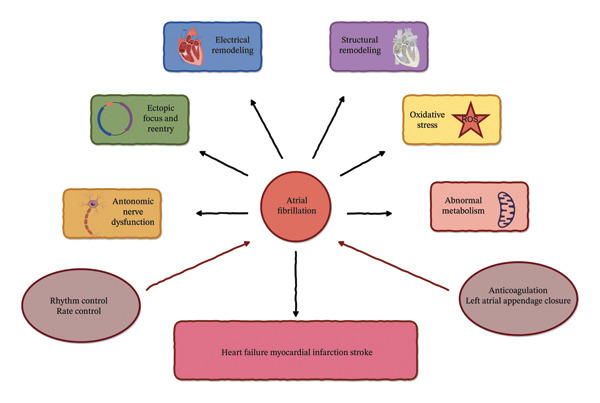
Atrial fibrillation: pathophysiology, therapeutic management, and associated complications.

Atrial fibrillation (AF), one of the most prevalent clinical arrhythmias, affects 1%–4% of the global population, with its prevalence continuing to rise due to population aging and increasing cardiovascular risk factors such as hypertension and obesity [1]. AF significantly elevates risks of stroke, heart failure with reduced ejection fraction (HFrEF), cardiomyopathy, acute coronary syndrome, cardiovascular mortality, myocardial infarction, and dementia [2–5]. It substantially impairs quality of life through symptom burden and psychological distress [6], while imposing substantial healthcare burdens. The pathophysiological alterations in AF encompass oxidative stress, structural remodeling (including fibrosis and atrial dilation), electrical remodeling driven by potassium/calcium/sodium channel abnormalities that shorten action potential duration (APD) and promote conduction heterogeneity along with reentrant circuit formation, autonomic dysfunction, metabolic dysregulation, ectopic activation, and reentry [7, 8]. Persistent AF differs mechanistically from paroxysmal AF, characterized by more severe structural remodeling and maladaptive ion channel alterations that establish a self‐perpetuating cycle of electrical remodeling and calcium handling dysregulation, thereby promoting AF maintenance and progression. Clinical management primarily encompasses rate/rhythm control, oral anticoagulation, and left atrial appendage closure—strategies wherein sinus rhythm restoration improves survival, quality of life, and ventricular function and reduces heart failure hospitalizations [8] (Figure 1). Consequently, the 2024 expert consensus statement by EHRA/HRS/APHRS/LAHRS on catheter and surgical ablation for AF [3], alongside the 2024 AHA/ACC/HRS guidelines [9] and EACTS guidelines [10], unanimously endorse catheter ablation techniques—specifically radiofrequency ablation and cryoballoon ablation—as first‐line therapeutic strategies in contemporary clinical practice. Nevertheless, contemporary ablation strategies for persistent AF remain suboptimal, with success rates approximating 50% [11]. High recurrence rates following initial catheter ablation—particularly in patients with advanced structural remodeling, chronic kidney disease, or hyperthyroidism—frequently necessitate repeat ablation or cardioversion [12]. While oral anticoagulation and early rhythm control improve prognosis by preventing stroke, cardiovascular mortality, heart failure hospitalization, and acute coronary syndromes, conventional antiarrhythmic drugs (e.g., sodium/potassium channel blockers) face substantial clinical challenges in persistent AF management due to single‐target mechanisms, ventricular adverse effects (including proarrhythmic potential), and limited efficacy in structural heart disease [13].

Although antiarrhythmic drugs modulate potassium/calcium/sodium channels or β1‐adrenoceptors to manage AF, their adverse effects—including ventricular arrhythmias induced by propafenone, sotalol [14], and ibutilide [15], along with amiodarone‐associated interstitial lung disease, thyroid dysfunction, and bradycardia [16]—significantly compromise patient outcomes. Consequently, exploring safer therapeutic alternatives is imperative. Natural compounds have garnered significant scientific interest in antiarrhythmic research due to their pleiotropic effects, low toxicity, and favorable safety profiles with minimal proarrhythmic risk, demonstrating considerable promise in both clinical applications and experimental investigations [17, 18]. Recent studies reveal that plant‐derived bioactive compounds—such as acacetin and tanshinone IIA—exhibit marked atrial selectivity and potent antiarrhythmic efficacy by selectively modulating atrial‐specific currents (e.g., IKur, IKACh), rectifying calcium dysregulation, and harnessing multifaceted effects including antioxidant and antifibrotic activity. This review systematically synthesizes core ion channel abnormalities in persistent AF, centering on natural compounds with validated antiarrhythmic efficacy to provide in‐depth mechanistic dissection of their precise targets and pathways for modulating channel function and blocking electrical remodeling—with the goal of establishing a theoretical foundation and pioneering clinical translation framework for developing highly safe, multitarget‐synergistic antiarrhythmic agents, while advancing pharmacologically active constituents from traditional botanicals as evidence‐based complementary therapeutics.

## 2. Ion Channel Abnormalities in Persistent AF

### 2.1. Potassium Channel Abnormalities and Electrical Remodeling

Cardiac potassium channels are categorized into voltage‐gated types (including IKur, IKr, Ito, IKs) and ligand‐gated types (including IK1, IK‐ACh, sarcKATP, mitoKATP, SK4). Atrial‐specific currents are predominantly mediated by IKur and IK‐ACh, whose functional abnormalities predispose to atrial arrhythmogenesis and play pivotal roles in AF pathogenesis and progression [19]. APD is primarily governed by repolarizing potassium currents, including Ito, IKur, and IK1 [20]. Abnormal enhancement of outward potassium currents such as IK1 and IK‐ACh markedly abbreviates APD, accelerating myocardial repolarization to directly facilitate reentrant AF formation [21]. During AF electrical remodeling, compensatory upregulation of IK1 and IK‐ACh currents mitigates intracellular calcium overload, while vagal activation potentiates IK‐ACh current—eliciting progressive APD abbreviation and reinforcing reentry pathway stability [13]. Molecular investigations reveal that downregulation of microRNA‐26 and ‐1 in AF patients augments Kir2.1 channel expression, driving enhanced IK1 current density to establish sustained atrial reentry [22]. Concurrently, gain‐of‐function mutations in KCNQ1—encoding the α‐subunit of IKs channels—create arrhythmogenic substrates by abbreviating atrial APD and reducing effective refractory period (ERP), thereby underpinning AF reentrant mechanisms [23].

Despite arrhythmogenic potential from extra‐cardiac factors like oxidative stress, the core mechanism of AF remains fundamentally centered on atrial potassium current remodeling—evolving from ion channel dysfunction to impaired electrical conduction, ultimately establishing self‐perpetuating reentrant circuits [24].

### 2.2. Calcium Handling Dysregulation and Triggered Activity

**FIGURE 2:**
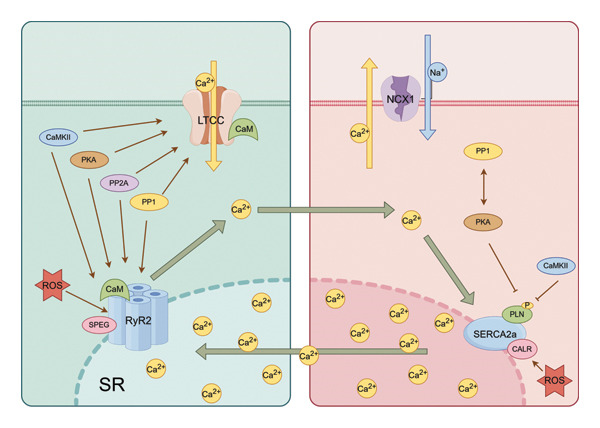
Sarcoplasmic reticulum (SR) serves as the major intracellular calcium (Ca^2+^) storage organelle. Its calcium leakage and spontaneous Ca^2+^ release events (SCaEs) are primarily caused by dysfunction of the cardiac ryanodine receptor type 2 (RyR2) channel or SR calcium overload. Dysregulation of RyR2 may result from increased expression, hyperphosphorylation—such as that induced by elevated calcium/calmodulin‐dependent protein kinase II (CaMKII) activity or impaired targeting of protein phosphatase‐1 (PP1)—as well as oxidation due to elevated reactive oxygen species (ROS). SR calcium overload can occur due to enhanced activity of SR calcium ATPase 2a (SERCA2a) or increased intracellular sodium concentration, the latter reducing calcium extrusion via the sodium/calcium exchanger type 1 (NCX1). Ca^2+^ influx triggers further Ca^2+^ release from the SR through RyR2 in a process known as calcium‐induced calcium release (CICR). The amount of Ca^2+^ released determines the amplitude of the calcium transient and influences the strength of myocardial contraction. SERCA2a, a key macromolecular complex responsible for Ca^2+^ reuptake into the SR, is allosterically regulated by its inhibitory subunit phospholamban (PLN). Phosphorylation of PLN by PKA and CaMKII relieves this inhibitory effect, facilitating rapid calcium resequestration. Under oxidative stress, calreticulin (CALR) may promote the inactivation and degradation of SERCA2a. Furthermore, similar to RyR2, the L‐type calcium channel (LTCC) is regulated by kinases—including PKA and CaMKII—as well as phosphatases such as PP1 and PP2A, which collectively modulate channel gating and its phosphorylation status.

Cardiac calcium channels are primarily categorized into high‐voltage‐activated types (L, P/Q, N, R) and low‐voltage‐activated types (T), where L‐type calcium channels (LTCCs) (predominantly Cav1.2) mediate ICa,L current to drive excitation‐contraction coupling and generate the action potential plateau phase [25, 26]. AF pathogenesis fundamentally hinges on calcium dysregulation, wherein under rapid pacing or AF conditions, sustained ICa,L attenuation shortens atrial APD—a compensatory electrical remodeling response that mitigates intracellular Ca^2+^overload [27–29]. This imbalance exacerbates calcium dysregulation through multilevel molecular pathways. Key mechanisms include dysregulated L‐type channel‐mediated calcium‐induced calcium release (CICR), RyR2 hyperactivation, Ca^2+^/calmodulin‐dependent protein kinase II (CaMKII) overexpression, or SPEG downregulation, collectively initiating diastolic calcium leakage that culminates in diminished calcium transient amplitude, delayed calcium decay, and enhanced spontaneous calcium elevations (SCaEs). Concurrently, CaMKII pathway activation further suppresses ICa,L current [30], reducing LTCC‐Cav1.2 expression while enhancing NCX1 activity—thereby increasing sodium‐calcium exchange current (INCX) and elevating intracellular sodium concentration. This process synergizes with augmented SERCA2a activity to induce sarcoplasmic reticulum (SR) calcium overload, ultimately triggering arrhythmias via delayed afterdepolarization (DAD) and early afterdepolarization (EAD) [31–34] (Figure 2). Despite regulatory involvement by calpain and protein phosphatase 2A [35], T‐type current remains comparatively stable. Ultimately, disordered intracellular Ca^2+^ signaling markedly potentiates atrial ectopic excitation and reentrant activity, inducing electrophysiological instability that sustains a self‐perpetuating cycle of AF.

### 2.3. Sodium Channel Dysfunction and Conduction Impairments

Cardiac sodium channels (predominantly the Nav1.5 subtype encoded by SCN5A) activate at membrane potentials between −70 and −60 mV, mediating the fast sodium current (INa) to trigger rapid phase 0 depolarization—serving as the cornerstone of excitation‐contraction coupling and atrial/ventricular impulse propagation [36, 37]. Dysfunction in these channels directly potentiates atrial electrical instability, where ectopic foci readily establish reentrant circuits that initiate AF [38]. Pathological alterations in sodium channels during AF progression exhibit bidirectional effects. On the one hand, SCN5A mutations [39] or reactive oxygen species (ROS) depress peak INa while enhancing late sodium current (INa, L) through PKC/CaMKII pathways—inducing conduction blockade, prolonged APD, and EADs that trigger arrhythmias [40]. Conversely, diminished INa intensity synergizes with gap junction impairments in low resting potential regions (e.g., pulmonary vein reentrant sites), significantly decelerating conduction velocity (CV) [41], while compensatory upregulation of sodium‐calcium exchanger (NCX) activity converts SR calcium release into strong inward currents (reverse mode)—initiating triggered premature beats by reaching threshold potential [42].

These mechanisms ultimately converge on the core pathway of energy metabolism derangement: mitochondrial dysfunction engenders energy deficiency and oxidative stress, synergizing with dysregulated sodium/calcium handling [43] to establish a bidirectional Na^+^–Ca^2+^ vicious cycling—ranging from INa conduction attenuation to NCX‐mediated calcium‐dependent electrical disturbances—that continuously propels atrial electrical remodeling toward decompensated AF phenotypes.

Persistent AF is sustained through a self‐perpetuating cycle driven by synergistic electrical and structural remodeling originating from ion channel dysfunction. Conventional antiarrhythmic agents exhibit proarrhythmic liability due to ventricular ionic current suppression, underscoring an exigent clinical demand for safer atrial‐selective interventions. Phytochemically derived bioactive compounds—leveraging their polypharmacological attributes—are emerging as transformative candidates transcending the current therapeutic limitations. Through targeted modulation of atrium‐predominant ionic currents, these compounds selectively prolong atrial ERP while concurrently rectifying calcium dysregulation and enhancing conduction fidelity—demonstrating potent antiarrhythmic efficacy without compromising ventricular safety. Furthermore, their synergistic antioxidant and antifibrotic actions halt atrial remodeling processes, establishing innovative disease‐modifying approaches for comprehensive AF management.

## 3. Therapeutic Modulation by Botanically Derived Bioactive Compounds

### 3.1. Acacetin

Acacetin, a flavone subtype of flavonoids, is a mono‐methoxy flavonoid compound distributed in diverse plant species—including *Robinia pseudoacacia*, *Chrysanthemum morifolium*, *Saussurea involucrata*, and others—predominantly as aglycone or glycoside forms [44, 45]. This naturally occurring flavonoid has demonstrated significant pharmacological efficacy, encompassing anti‐inflammatory, anticancer, antiobesity, antidiabetic, neuroprotective, and cardioprotective activities [44].

Employing whole‐cell patch‐clamp techniques, Li et al. [46, 47] demonstrated that acacetin potently inhibits human atrial IKur (ultrarapid delayed rectifier potassium current) and Ito (transient outward potassium current) with IC50 values of 3.2 and 9.2 μmol/L, respectively. Within the 3–10 μmol/L concentration range, this compound effectively suppressed carbachol‐induced IK, ACh (acetylcholine [ACh]‐activated potassium current) in guinea pig atrial myocytes, thereby prolonging vagally mediated atrial APD and ERP. Critically, acacetin at 30–100 μmol/L exhibited no inhibitory effects on sodium current (INa), L‐type calcium current (ICa, L), or inward rectifier potassium current (IK1) in ventricular myocytes—confirming its atrial‐selective pharmacodynamic profile and favorable safety margin. Particularly noteworthy is acacetin’s potent preferential inhibition of atrial‐specific IKur—which exhibits exclusive atrial expression with no ventricular counterpart—while concurrently targeting Ito, IK, ACh, and small‐conductance Ca^2+^‐activated K^+^ currents (SKCa) with IC50 values below 10 μmol/L [48, 49]. This multitarget engagement of atrium‐selective ion channels positions acacetin as a prototypic candidate for developing atrial‐selective antiarrhythmic agents, thereby circumventing the proarrhythmic liabilities of conventional drugs stemming from ventricular ion channel suppression.

Studies demonstrate that selective IK, ACh inhibitors effectively prevent vagally mediated AF [50, 51]. As a multitarget inhibitor, acacetin specifically suppresses IKur, Ito, and IK, ACh channels, significantly prolonging atrial APD and ERP following intraduodenal administration in anesthetized canines—mediating potent anti‐AF efficacy. Critically, acacetin exhibits superior atrial selectivity without inducing QT interval (QTc) prolongation compared to sotalol. Toxicological evaluation confirmed exceptional safety, with no mortality observed at the maximum tolerated dose (900 mg/kg) during oral administration in mice [47]. These findings collectively underscore acacetin’s clinical translational value as a novel atrial‐selective antiarrhythmic agent.

Acacetin mediates antiarrhythmic effects by specifically blocking hKv4.3 (mediating Ito) and hKv1.5 (mediating IKur) channels, exhibiting use‐ and frequency‐dependent blockade characteristics. Molecular mechanistic studies reveal that acacetin interacts with the P‐helix (T366A, T367A) and S6 segment (V392A, I395A, V399A) residues of hKv4.3 [52], while concurrently targeting the S6 domain residues (V505A, I508A, V512A) of hKv1.5 [53], generating steric hindrance effects. Furthermore, combination therapy with sodium channel blockers enhances anti‐AF efficacy through cooperative multichannel inhibition [54]. This dual Kv1.5/Kv4.3 blockade mechanism provides a theoretical foundation for developing precision antiarrhythmic strategies leveraging spatiotemporal ion channel properties.

Furthermore, emerging evidence suggests that acacetin’s cardioprotective effects extend beyond ion channel modulation to involve oxidative stress regulation—a critical pathophysiological contributor to cardiovascular diseases. By upregulating myocardial antioxidant enzymes (e.g., superoxide dismutase [SOD] and glutathione peroxidase [GSH‐Px]) while reducing ROS levels, acacetin mitigates oxidative damage to cardiomyocytes. This mechanism potentially expands the mechanistic rationale for its therapeutic application in AF [55]. Future investigations should elucidate crosstalk between acacetin and key signaling pathways (e.g., calcium homeostasis, MAPK cascades) and refine dosing regimens (optimizing concentrations and administration intervals) to enhance therapeutic efficacy. Such interrogation will further delineate acacetin’s polypharmacology and strengthen its translational foundation.

### 3.2. Puerarin

Puerarin, a naturally occurring isoflavone derivative extracted from the traditional Chinese herb *Pueraria lobata* [56], demonstrates multifaceted pharmacological activities including—but not limited to—anti‐inflammatory [57], antioxidant [58], antiapoptotic [59], neuroprotective [60], antiatherosclerotic [61], and antiarrhythmic [62] effects.

Electrophysiological studies reveal that Puerarin significantly enhances electrophysiological stability in hypertrophied cardiomyocytes by mitigating the decay of maximum depolarization rate and augmenting peak sodium current (INa) amplitude, thereby reducing arrhythmia susceptibility [63]). Furthermore, millimolar‐range concentrations of Puerarin positively shift resting membrane potential while potently inhibiting inward rectifier K^+^ channels (Kir2.1/Kir2.3) and directly suppressing Kv7.1‐mediated slow delayed rectifier potassium current (IKs) in a PKA‐independent manner. Separately, micromolar concentrations of Puerarin concentration‐dependently prolong APD in isolated rat cardiomyocytes [64].

Emerging evidence underscores a critical link between maladaptive ion channel remodeling and fibrotic disease progression [65]. Functioning as a pluripotent natural compound, puerarin significantly downregulates key signaling molecules within the TGF‐β/Smad3 pathway, consequently attenuating profibrotic gene transcription [66]. This dual mechanism not only attenuates fibrotic progression but also alleviates ion channel remodeling‐induced cellular dysfunction. These findings elucidate puerarin’s capacity to orchestrate multitargeted ion channel remodeling against AF, providing foundational experimental evidence for its electrophysiological modulation of AF substrates.

### 3.3. Quercetin

Quercetin, a naturally occurring polyhydroxylated flavonoid, is present as glycosides in vegetables and fruits, as well as in numerous traditional Chinese medicines such as *Astragalus membranaceus*, *Ephedra sinica*, and *Eucommia ulmoides*. It exhibits a broad spectrum of biological activities, including antioxidant [67], anti‐inflammatory [68], antihypertensive [69], cardioprotective [70], and antiarrhythmic [40] effects.

Functioning as a calcium channel blocker, quercetin exerts antiarrhythmic effects by inhibiting myocardial calcium influx and potassium efflux, reducing calcium chloride‐induced automaticity and mitochondrial membrane potential. In isoproterenol‐induced myocardial ischemia models, quercetin concentration‐dependently suppresses L‐type calcium current (ICa‐L), characterized by an upward shift of the current–voltage curve and a leftward shift of the activation/inactivation curves, while concurrently inhibiting cell contraction amplitude and calcium transients, thus demonstrating cardioprotective effects [71].

Studies reveal atrial‐specific dysregulation of miRNA profiles in AF patients, where Wang et al. documented significant upregulation of miR‐513b, miR‐1181, and miR‐887 alongside downregulation of miR‐135b, miR‐100, and miR‐892a [72]. Mechanistically, quercetin upregulates miR‐135b expression by suppressing the TGF‐β/Smads signaling axis, subsequently downregulating cyclin D1, α‐SMA, and collagen‐synthesis mediators, effectively attenuating isoproterenol‐induced atrial fibrosis and fibrillation progression [72]. Furthermore, Hu et al. demonstrated that quercetin activates autophagy via the miR‐223‐3p/FOXO3 molecular axis, evidenced by concomitant increases in ATG7 and LC3B‐II expression with reduced p62/SQSTM1 levels, thereby inhibiting myocardial fibrosis and reversing AF‐associated atrial structural remodeling [73].

### 3.4. Naringin

Naringin, a flavonoid extracted from citrus fruits, is derived from the peel of *Citrus paradisi* (Rutaceae) [74]. Its aglycone form is termed naringenin. Structurally, this compound belongs to the flavanone subclass of flavonoids, featuring neohesperidose—a disaccharide linked via a glycosidic bond at the C7 position. Naringin demonstrates broad‐spectrum bioactivities and pharmacological properties with therapeutic applications in oncology [75], diabetes mellitus [76], neurodegenerative disorders [77–79], metabolic syndrome [80], osteoporosis [81], and cardiovascular pathologies.

Semaphorin 3A (Sema3A) deficiency contributes to AF progression by promoting endothelial‐mesenchymal transition (EndMT) in atrial fibrosis. Utilizing human atrial tissues, isolated atrial endocardial endothelial cells (AEECs), and cardiac‐specific TGF‐β‐overexpressing transgenic mice, Lai et al. [82] demonstrated that EndMT markers (Twist), proliferation markers (PCNA), and endothelial markers (CD31) co‐drive endocardial fibrosis in AF patients and model mice. Naringenin (a Sema3A activator) specifically suppresses AEEC proliferation and inhibits EndMT markers through Sema3A upregulation, as validated by siRNA knockdown experiments. In TGF‐β transgenic mice, naringenin ameliorates AF and endocardial fibrosis by activating Sema3A expression, inhibiting EndMT, reducing fibrotic deposition, and attenuating electrical remodeling susceptibility, suggesting its potential as a therapeutic candidate for targeted AF intervention.

Further research demonstrates that naringin functions as a multichannel blocker, exerting antiarrhythmic effects through modulating transmembrane ion fluxes [83]. At the cellular level, naringin inhibits L‐type calcium current (ICa‐L; IC50 = 508.5 μM), late sodium current (INa‐L; IC50 = 311.6 μM), and peak sodium current (INa‐P) while simultaneously prolonging delayed rectifier potassium current (IK) and transient outward potassium current (Ito). These actions collectively shorten APD, reduce maximal depolarization rate, and suppress both EADs induced by Anemonia sulcata toxin II (ATX‐II) and DADs triggered by calcium chloride. In ex vivo heart models, naringin effectively reduced both the incidence and duration of ATX‐II‐induced arrhythmias.

Beyond its regulation of ion signaling pathways, naringin’s cardioprotective effects may involve modulation of oxidative stress mediators. Studies demonstrate that naringin reduces ROS generation in cardiomyocytes [84, 85]. This ROS attenuation maintains intracellular homeostasis by alleviating oxidative damage, while simultaneously modulating calcium signaling through complementary crosstalk with CaMKII and PKA pathway inhibition [86, 87]. Such dual mechanism collectively enhances myocardial protection.

### 3.5. Matrine

Matrine (MAT), a naturally occurring quinolizidine alkaloid widely distributed in traditional Chinese medicinal herbs such as *Sophora flavescens* and *Eucommia ulmoides* [88], demonstrates extensive pharmacological activities including antiarrhythmic, antifibrotic, antitumor, anti‐inflammatory, analgesic, and antiviral properties [89]. Experimental evidence reveals that 100 μmol/L MAT competitively inhibits ouabain binding to LTCCs, reducing ICa‐L current in guinea pig ventricular myocytes (approximately 80% decrease in calcium transient amplitude), thereby reversing abnormal APD prolongation and alleviating calcium overload [90]). Pathological cardiac fibrosis impairs myocardial contractility transmission, disrupts normal electrical conduction, and contributes to systolic/diastolic dysfunction with potentially fatal arrhythmias [91].

In cardiac fibrosis models, matrine administered via gavage at 100 mg/kg/d for 4 weeks upregulated Cav1.2 protein expression in murine atrial myocytes, improving left atrial conduction and reducing AF duration [92]. Mechanistic studies revealed that 200 μmol/L matrine suppressed P38 MAPK signaling pathway activity in cardiomyocytes, thereby inhibiting fibroblast proliferation, migration, and differentiation to ameliorate cardiac remodeling [93]. Furthermore, Zhang et al. [94] integrated clinical sample analysis with transverse aortic constriction (TAC) mouse models, demonstrating that matrine attenuates pressure overload‐induced AF through inhibition of the Wnt3a/β‐catenin signaling pathway. These findings collectively indicate matrine’s multitargeted modulation of calcium channel functionality, highlighting its potential as a therapeutic candidate for antiarrhythmic drug development.

### 3.6. Berberine

Berberine, a widely used isoquinoline alkaloid primarily extracted from *Coptis chinensis*, is clinically prescribed for treating infections, diabetes mellitus, and cardiovascular disorders [95, 96]. Previous investigations have classified berberine as a class III antiarrhythmic agent, suggesting its potential suitability for AF management [97]. Electrophysiological studies in pentobarbital‐anesthetized adult rabbits demonstrated that berberine prolongs both the RR interval and atrial ERP, significantly reducing AF induction rate while enhancing AF termination efficiency. This compound effectively reversed ACh‐induced shortening of APD in atrial myocytes and potently suppressed ACh‐triggered AF in the experimental models [98].

Berberine exerts potent antiarrhythmic effects through its significant antioxidant, anti‐inflammatory, and antiapoptotic activities, while simultaneously enhancing mitochondrial bioenergetics. Crucially, Berberine attenuates electrical remodeling by modulating multiple ion channels: it effectively regulates potassium currents (IK, IK1, IKr, IKs), calcium current (ICa), sodium current (INa), KATP channels, and the human ether‐a‐go‐go‐related gene (hERG) potassium channel. Additionally, Berberine upregulates inward rectifier potassium channels (Kir6.2 and Kir2.1), prolongs APD, and extends the ERP [99–107].

Further investigations suggest that berberine’s antiarrhythmic efficacy in AF may additionally involve modulation of intracellular calcium homeostasis. Elevated intracellular calcium concentrations are recognized as pivotal contributors to atrial electrophysiological remodeling [108]. By inhibiting CaMKII activity, berberine not only indirectly modulates inward rectifier potassium current (IK1) but also regulates calcium channel functionality, thereby preserving calcium homeostasis [109]. This mechanism likely underlies berberine’s documented improvements in atrial conduction heterogeneity among AF patients. Future research should explore berberine’s combinatorial potential with conventional antiarrhythmic drugs. Given its unique multitarget profile, synergistic effects may be achieved through coadministration with standard therapies, potentially enhancing AF treatment efficacy.

### 3.7. Resveratrol

Resveratrol, a natural polyphenol abundant in grapes, mulberries, and various botanicals [110], exhibits anti‐inflammatory, antioxidant, antiarrhythmic, and endothelium‐protective properties [111]. This compound prevents arrhythmias through modulation of sodium, potassium, and calcium channels [110, 112].

Studies confirm resveratrol and its derivatives modulate AF electrophysiology. In ex vivo rabbit AF models, acute resveratrol intervention reduced AF induction frequency (though not overall susceptibility) by decelerating basal heart rate, prolonging atrial ERP, and suppressing electrical CV [113]. Notably, structurally optimized resveratrol derivatives targeting Kv1.5 channels (pore‐forming subunits of ultra‐rapid delayed rectifier potassium current, IKur) demonstrated enhanced efficacy in canine atrial tachycardia models: enhanced binding affinity to Kv1.5 prolonged atrial ERP and shortened AF duration [114]. This resulted in improved cardiac mechanical function, reduced myocardial infarction area, and decreased incidence of ischemia‐induced arrhythmias [115, 116].

Resveratrol’s cardioprotective effects may additionally involve modulation of apoptotic signaling pathways. Research confirms that SIRT1 activation suppresses the mitochondrial apoptotic pathway, reducing cardiomyocyte apoptosis [117]. By activating SIRT1, resveratrol inhibits apoptosis in experimental persistent AF models, thereby enhancing cardiac protection [118]. Cao et al. [119] employed high‐frequency electrical stimulation of HL‐1 atrial myocytes to establish an AF model, identifying optimal concentrations via CCK‐8/flow cytometry. Resveratrol significantly reversed AF‐induced mitochondrial dysfunction and preserved ultrastructural integrity. Gain‐of‐function experiments demonstrated that resveratrol ameliorates AF‐associated metabolic disturbances by enhancing SIRT3 activity to regulate acetylation modifications of key metabolic enzymes [119].

### 3.8. Curcumin

Curcumin, a natural polyphenolic compound extracted from dried rhizomes of *Curcuma longa*, exhibits anti‐inflammatory, antioxidant, and antifibrotic properties [120]. Using whole‐cell patch‐clamp techniques and multichannel synchronous recording systems, Lv et al. [121] demonstrated that curcumin functions as a multi‐ion channel blocker with preferential inhibition of late sodium current (INa.L), exerting antiarrhythmic effects at cellular and organ levels through multi‐ion current modulation. Specifically, 30‐μmol/L curcumin significantly shortened APD at 50% and 90% repolarization (APD50 and APD90) by 17% and 7%, respectively. It dose‐dependently suppressed INa.L, transient sodium current (INa.T), L‐type calcium current (ICa.L), and rapid delayed rectifier potassium current (IKr), with respective IC50 values of 7.53, 398.88, 16.66, and 9.96 μmol/L. Notably, curcumin exhibited 53‐fold greater potency against INa.L compared to INa.T [121].

Studies demonstrate that in hypertensive p300‐knockout mouse models, AF incidence decreases due to impaired NLRP3 inflammasome activation [122]. Further in vitro experiments reveal that p300 overexpression directly binds NF‐κB, thereby activating the NLRP3 inflammasome and upregulating the atrial ultrarapid delayed rectifier potassium current (IKur), which mediates hypertension‐associated AF susceptibility [122]. Concurrently, substantial evidence indicates that the p300/p53 axis promotes senescence‐related atrial fibrosis by regulating plasminogen activator inhibitor‐1 (PAI‐1) expression, consequently accelerating AF progression [123–125]. As a p300 inhibitor, curcumin significantly attenuates such atrial electrical remodeling and fibrotic changes [122, 124], with multiple studies confirming its therapeutic effects against AF through amelioration of myocardial fibrosis [126, 127].

### 3.9. Tanshinone IIA

Tanshinone IIA, a lipophilic diterpenoid compound isolated from the dried roots and rhizomes of *Salvia miltiorrhiza* [128], represents a promising natural cardioprotective agent with established anti‐inflammatory properties [129]. Contemporary pharmacological investigations have documented that tanshinone IIA exhibits multiple therapeutic effects, including antiatherosclerotic [130], antimyocardial ischemic [131], antiarrhythmic [128], and cardiomyocyte antiapoptotic activities [132].

**FIGURE 3:**
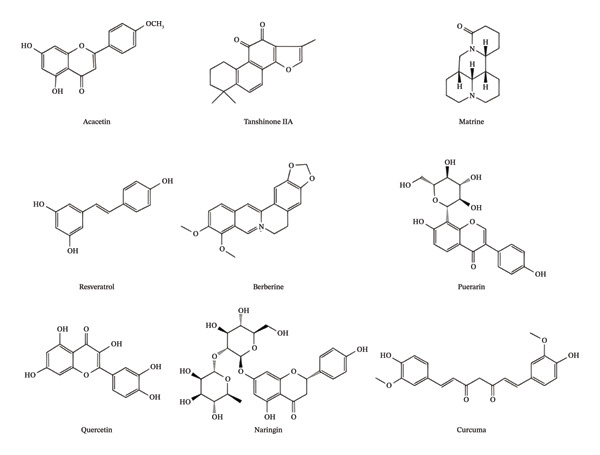
Natural medicine chemical structural formula.

In a clinical trial by Yang et al. [133], 300 post‐PCI patients were randomized into three treatment groups: Group A (alprostadil monotherapy), Group B (tanshinone IIa injection monotherapy), and Group C (combination therapy). After 7 days of treatment, Group C demonstrated significantly superior cardiac function parameters—including left ventricular end‐diastolic dimension (LVEDD), left ventricular ejection fraction (LVEF), and E/A ratio—along with enhanced myocardial contrast echocardiography metrics (peak intensity rise rate [Aβ], myocardial perfusion grading) compared to monotherapy groups (*p* < 0.05). During the 12‐month follow‐up, Group C exhibited significantly reduced incidence of major adverse cardiac events (MACE), encompassing malignant arrhythmias, recurrent heart failure, myocardial reinfarction, and all‐cause mortality. These findings confirm that tanshinone IIA injection combined with alprostadil effectively improves post‐PCI cardiac function, increases myocardial perfusion, and reduces malignant arrhythmia incidence [133]. In a study investigating chronic heart failure with AF prevention and treatment, tanshinone IIA moderately increased atrial conduction time, ERP, and post‐repolarization refractoriness in isolated rabbit hearts, thereby reducing AF inducibility. This effect is attributed to its inhibition of late sodium current (INa.L) and calcium overload [134]. Further research demonstrates that tanshinone IIA’s modulation of cardiomyocyte electrophysiology extends beyond ERP prolongation. It alleviates intracellular Ca^2+^ overload, reducing cellular excitability and arrhythmia susceptibility. Wang et al. revealed that tanshinone IIA suppresses intracellular calcium signaling and cell adhesion pathways during early‐stage myocardial ischemic injury, conferring cardiomyocyte protection [135]. Additional experiments confirmed that tanshinone IIA directly inhibits Ca^2+^ influx through LTCCs in vascular smooth muscle cells and dilates isolated rat coronary arteries [136]. Salvianolic acid B, the principal bioactive component among water‐soluble phenolic acids in *Salvia miltiorrhiza*, has been demonstrated to mitigate calcium overload‐associated electrical remodeling through direct inhibition of calcium current [137] (Figure 3).

### 3.10. Ginsenosides

Ginsenosides, glycosidic compounds derived from *Panax ginseng* and *Panax notoginseng*, exhibit significant cardiovascular activity. Studies confirm that total ginsenosides inhibit L‐type calcium current and reduce intracellular calcium concentration [138]. Notably, specific ginsenosides including Re, Rg1, Rb1, Rg2, and Rg3 function as LTCC blockers. Ginsenoside Rb1 exerts pharmacological effects by modulating sodium, potassium, and calcium channels in neuronal membranes [139]. Furthermore, Liu et al. demonstrated that ginsenoside Rb1 suppresses INa and ICa.L in rabbit ventricular myocytes subjected to ischemia‐reperfusion injury, reduces action potential amplitude and maximum upstroke velocity, eliminates high‐calcium‐induced DADs, inhibits calcium overload, and ultimately protects cardiomyocytes [140]. Ginsenoside Rg2 pretreatment suppresses phosphorylation of Ca^2+^/calmodulin‐dependent protein kinase IIδ (CaMKIIδ), reduces LTCC‐mediated Ca^2+^ influx, and significantly decreases mortality, duration, and incidence of malignant arrhythmias in calcium chloride‐induced arrhythmia rat models [141]. Liu et al. demonstrated that ginsenoside Rg3 improves calcium cycling and suppresses calcium overload in cardiomyocytes from mice with pathological hypertrophy and heart failure by enhancing SERCA2a activation [142]. Through modulation of calcium channel activity, ginsenosides reduce Ca^2+^ influx, thereby alleviating intracellular calcium overload. This regulatory mechanism maintains physiological cardiomyocyte contraction‐relaxation dynamics and potentiates their therapeutic efficacy against AF.

**FIGURE 4:**
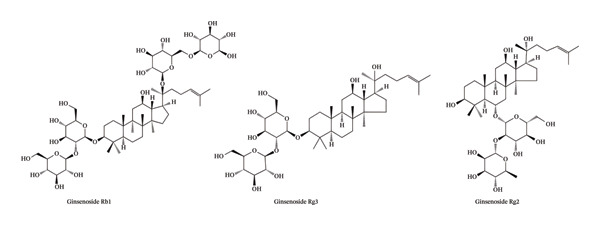
Ginsenoside.

Ginsenoside Rb1 may further exert cardioprotective effects by modulating cardiomyocyte energy metabolism. Studies demonstrate that it enhances mitochondrial function, increases ATP production, and thereby provides sufficient energy supply for normal physiological activities of cardiomyocytes [143, 144]. Furthermore, rigorously validated through yeast NDH bypass assays, molecular docking, and surface plasmon resonance analysis, Rb1 binds to the ND3 subunit of mitochondrial complex I, locking it in an inactive conformation. This mechanism specifically inhibits complex I‐dependent respiration (without affecting complexes II/IV), reduces mitochondrial ROS generation during reperfusion, and consequently improves cardiac function while suppressing fibrosis [139]. This mode of action may consequently ameliorate energy metabolism disorders in AF patients’ cardiomyocytes, mitigating AF‐associated pathophysiological alterations (Figure 4).

Plant‐derived natural compounds modulate AF pathogenesis through multidimensional mechanisms. These bioactive constituents collectively demonstrate the therapeutic significance of natural products in regulating transmembrane ion fluxes, ameliorating electrical remodeling (via APD/ERP prolongation), suppressing fibrotic progression, and maintaining calcium homeostasis, thereby highlighting their unique advantages in achieving synergistic multitarget interventions (Tables 1).

**TABLE 1:** Plant‐derived bioactive compounds: source plant, categories, activities, and ion channel targeting.

Compound	Activity	Source plant	Categories	Targeting ion channels
Acacetin	Prolonged APD, Prolonged ERP	*Chrysanthemum morifolium* Ramat. *Saussurea involucrata* (Kar. et Kir.)Sch.‐Bip.	Flavonoid	↓IKur, ↓Ito, ↓IK‐ACh

Puerarin	Prolonged APD, Increase the peak amplitude of INa	*Pueraria lobata* (Willd.)Ohwi	Flavonoid	↓IK1, IKs, ↑INa

Quercetin	Inhibits Ca^2+^ overload	*Ephedra sinica* Stapf. *Eucommia ulmoides* Oliv.	Flavonoid	↓LTCC

Naringin	Prolonged APD, Inhibits DADs, Inhibits EADs, Inhibits CaMKII activity	*Citrus aurantium* L.	Flavonoid	↓IK, ↓Ito, ↓LTCC, ↓INaLate

Matrine	Prolonged APD, Inhibits Ca^2+^ overload	*Sophora flavescens* Ait. *Eucommia ulmoides* Oliv.	Alkaloid	↓LTCC

Berberine	Prolonged APD, Prolonged ERP, Inhibits Ca^2+^ overload, Inhibits CaMKII activity	*Coptis chinensis* Franch.	Alkaloid	↓IK1, IKr, IKs, IK‐ACh, ↓KATP, ↓LTCC, ↑INa

Resveratrol	Inhibits DADs, Prolonged ERP, Inhibits Ca^2+^ overload, Slow down the basal heart rate	*Reynoutria japonica* Houtt.	Polyphenol	↓IKur, ↓Ito, ↓LTCC

Curcuma	Prolonged APD, Prolonged ERP, Inhibits Ca^2+^ overload	*Curcuma longa* L.	Polyphenol	↓IKur, IKr, ↓LTCC, ↓INaLate

Tanshinone IIA	Prolonged APD, Prolonged ERP, Inhibits Ca^2+^ overload	*Salvia miltiorrhiza* Bge.	Diterpene	↓INaLate, ↓LTCC

Ginsenoside	Inhibits DADs, Inhibits Ca^2+^ overload, Inhibits CaMKII activity	*Panax ginseng* C. A. Mey. *Panax notoginseng*(Burk.)F. H. Chen	Glycoside	↓LTCC

## 4. Conclusions

Persistent AF demonstrates significant complexity and dynamic evolution in its ion channel abnormalities, highlighting the limitations of single‐target interventions. Current evidence indicates that dysfunction in potassium, calcium, and sodium channels, coupled with dysregulation of associated signaling networks, collectively drive AF initiation and maintenance [33, 145]. Consequently, multitarget therapeutic strategies possess greater clinical potential.

Plant‐derived natural compounds have gained considerable recognition for their low toxicity and high efficacy. These agents exhibit multiconstituent, multitarget, and multipathway properties against AF, establishing them as novel therapeutic candidates. Diverse bioactive components—including flavonoids, alkaloids, and polyphenols—have been documented to exert antiarrhythmic effects by regulating ion channels, alleviating electrical remodeling, and suppressing fibrosis [146].

However, research on natural compounds for AF therapy currently faces multiple translational barriers. First, the antiarrhythmic efficacy of most bioactive constituents has been validated exclusively in animal models, lacking systematic clinical trial evidence. For instance, while tanshinone IIA demonstrates potent LTCC blockade in ex vivo cardiac models [147], its human pharmacokinetic profiles remain uncharacterized. Consequently, comprehensive evaluation of therapeutic potential must consider pharmacokinetic properties, bioavailability, and potential synergistic or antagonistic interactions. Second, the complexity of persistent AF presents substantial challenges—electrical remodeling (e.g., APD shortening) and structural remodeling (e.g., fibrosis) exhibit intertwined progression, yet current experimental models inadequately recapitulate the multifactorial pathology of human chronic AF. Moreover, the inherent polypharmacology of natural compounds, while potentially enhancing therapeutic outcomes, complicates mechanistic elucidation.

Future research should advance by synergizing modern pharmacological methodologies with traditional medical wisdom, meticulously investigating mechanisms of natural compounds while optimizing their structures to enhance target specificity and safety profiles. Concomitantly, rigorous preclinical evaluations and well‐designed clinical trials must be implemented to validate therapeutic efficacy and establish optimal dosing regimens. Integrating emerging technologies—including systems biology, network pharmacology, and artificial intelligence—could plausibly reveal multitarget regulatory networks of natural compounds, thereby accelerating their translational advancement from laboratory research to clinical implementation.

This review methodologically profiles natural active compounds targeting AF therapeutics through an ion channel‐centric lens, revealing underexploited multitarget resources whose mechanisms urgently demand comprehensive exploration and experimental validation. While these phytoactive compounds represent promising therapeutic avenues for AF, substantial clinical translation barriers persist. Consequently, strengthening the interface between fundamental research and clinical implementation remains imperative for developing evidence‐based multitarget strategies delivering precise, safe, and effective therapeutic outcomes.

## Author Contributions

Yize Zhai: conceptualization, literature screening, original draft writing, and data integration.

Na Shi: data collection, preliminary review, and critical revision of important content.

Wenzhuo Duan: figure preparation, reference organization, and data verification.

Jianfei Yang: study design, final review, funding acquisition, and corresponding author responsibility.

## Funding

No funding was received for this manuscript.

## Disclosure

All authors have reviewed and approved the final version of the manuscript.

## Conflicts of Interest

The authors declare no conflicts of interest.

## Data Availability

Data sharing is not applicable to this article as no datasets were generated or analyzed during the current study.
